# Supplementary figures and images for: Microevolution of the mexT and lasR Reinforces the Bias of Quorum Sensing System in Laboratory Strains of Pseudomonas aeruginosa PAO1
Source: Front Microbiol. 2022 Apr 12;13:821895. doi: 10.3389/fmicb.2022.821895 (PMC9041413; doi:10.3389/fmicb.2022.821895)

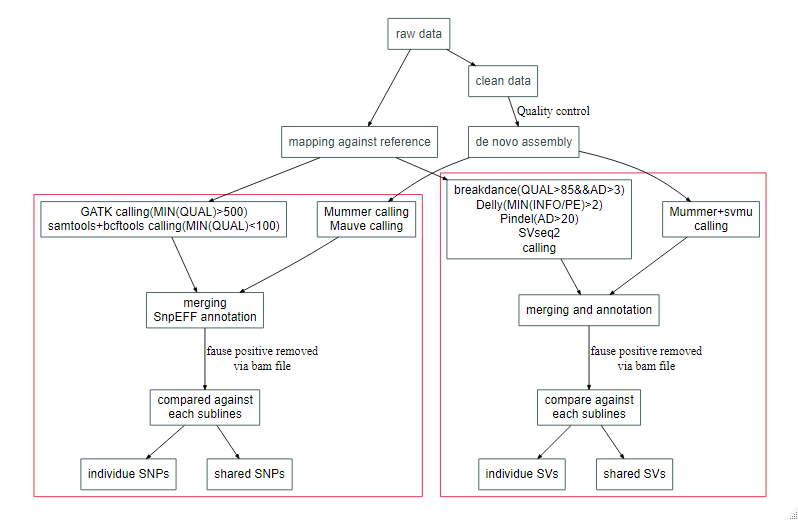

Supplement: Supplementary file 2 [file Image_1.TIF]

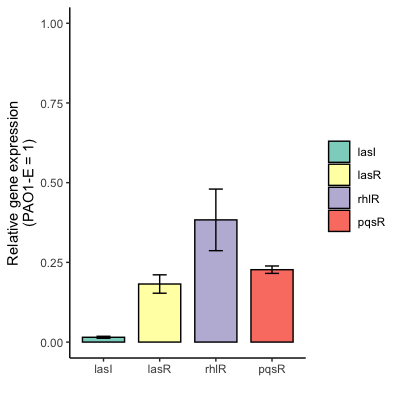

Supplement: Supplementary file 3 [file Image_2.TIFF]
